# American Medical Association

**Published:** 1852-06

**Authors:** 


// THE WESTERN JOURNAL.

Third Series.—Vol. IX.—No. VI.

LOUISVILLE, JUNE 1, 1852.

AMERICAN MEDICAL ASSOCIATION.

*Richmond, May 7th,* 1852.

Gentlemen—According to promise, I proceed to give you some account of the last meeting of the American Medical Association.

The Association met in this city, pursuant to adjournment, on Tuesday morning the 4th of May. Dr. Haxall, the Chairman of the Committee of Arrangements, read the names of the delegates present, as far as enrolled, and the several State delegations proceeded to select each a committeeman on nominations. Our friend Dr. Sutton, a delegate from the Medical Society of Kentucky, was appointed unanimously by the Kentucky delegation, and Dr. Eve, the only representative from Tennessee, acted as committeeman from that State.

The committee retired, and, during its absence, Dr. Moultrie delivered the annual address. It was a subject of general regret that he spoke in so low a tone of voice as to be entirely inaudible save to those who were very near to him.

After the address, the committee reported the following names for officers during the ensuing year:—President, Dr. J. B. Welford, of

Fredericksburg, Va.; Vice-Presidents, Drs. Jonathan Knight, of Connecticut, Jas. W. Thompson, of Delaware, T. Y. Simmons, of S. C., C. A. Pope, of Mo., and D. Francis Condie, of Pennsylvania, who were all unanimously elected. Dr. Wellford was conducted to the chair by a committee, and returned thanks in a few remarks which were very happy.

Dr. Sequard, of Paris, a young gentleman of some celebrity as an experimental physiologist, one of whose productions you have seen in the last number of the Philadelphia Medical Examiner, and Drs. Semmes and Berry, of the District of Columbia, were elected members by invitation; after which the Association adjourned till 4½ o'clock.

The first report read during the afternoon was from the Committee on Prize Essays, of which Dr. Hayward of Boston, was chairman. Dr. H. stated that fourteen essays had been offered, two of which were not examined by the committee, one having been received at too late a day, and the name of the author of the other having been made known to the committee. The distinguished gentleman further remarked, that after a very careful and patient examination of the several essays, the committee had awarded but a single prize, and that the first, no essay being deemed altogether worthy of the second. He thought it necessary to state, however, that among the essays offered, there were several besides that on which the prize had been conferred, worthy of honorable mention, though it was due the committee to say that, except this one, none, in their opinion, had "contributed to the increase of our knowledge or the enlargement of the boundaries of science."

The package containing the inscription corresponding to that borne by the prize essay was opened in presence of the Association, and found to be signed by Dr. Austin Flint, of Buffalo. The title of the essay was *The Variations of Pitch in Percussion and Respiration Sounds as a Means of Physical Diagnosis.* The announcement was received with much applause.

The committee reported in favor of St. Louis as the next place of meeting and a change of one of the secretaries to suit the locality. After which the Association adjourned.

Wednesday morning, as soon as the the Minutes had been read and approved, the secretary informed the Association that he enclosed copies of the preamble and resolutions adopted by the Association at their session of 1850–'51, relative to assimilated rank of the Medical Staff of the Army and Navy to the several departments, as directed by the resolutions, and from Dr. Harris, chief of the Bureau of Medicine and Surgery, he had received a letter approving of the course of the Association, which he proceeded to read.

Dr. Pinckney, of the Navy, asked leave to read a memorial which he had prepared to present to Congress on the subject of assimilated rank. Leave was granted, and the gentleman read a very excellent memorial, and followed it by an explanatory speech, which, save in its length, was not unacceptable to the Association.

Dr. Cox, of New York, offered resolutions approving the memorial of Dr. Pinckney by the Association, urging on Congress a calm and dispassionate consideration of them and speedy legislation concerning them. He felt confident the memorial would be granted; indeed he did not see how it could be longer refused. It was a matter of great interest to the profession, not only to those in the army and navy but to civilians also. He believed indeed that Congress would not allow the present session to close without defining clearly and definitely the relative rank of the Medical Staff of the Navy, and he wished the Secretary of this Association to be instructed to address a copy of the resolutions and Dr. Pinckney's memorial to the Secretary of the Navy, and President of the Senate and House of Representatives.

Dr. D. W. Yandell, of Ky., moved a reference of the resolutions to a committee of three, to be appointed by the president—which was carried.

Dr. Atkinson, of Va., offered the following:

*Resolved,* That we have listened with pleasure to Dr. Pinckney's able and eloquent remarks in vindication of the honor and interest of the profession, and that we will second his efforts to obtain justice from Congress by every means in our power. The resolution was adopted and referred to the same committee.

Dr. Simons, of Charleston, S. C., offered a preamble setting forth the state of emigrant ships as regards disease, and especially those arriving at the ports on the southern shores of the United States from California, and their not being provided with medical attendants. Dr. S. regarded this as deserving our attention as conservators of health, and thought it our duty to lay these facts before Congress. He therefore proposed that Congress be desired to require all vessels bringing steerage passengers to the United States to be provided with a surgeon, and that a committee be appinted to carry out the resolution and make suggestions in regard to the importance of the measure.

Dr. Wood, of Philadelphia, made a motion that the preamble and resolutions be laid on the table for the present; which was carried.

Dr. J. B. Flint, of Ky., proposed to alter the Constitution by substituting for the annual volume of Transactions now published, a quarterly journal which should contain the proceedings, be conducted under the auspices and considered the exponent of the views of the Association, on medical science, education and ethics.

Dr. Storer, of Boston, the Chairman of the Committee on Obstetrics which made its report at Charleston, wished to claim the indulgence of the Association for a few moments. He had found himself the subject of an anonymous and scurrilous attack in the April No. for 1852, of Nelson's Medical Journal. He desired it to be distinctly understood that he rose not as a private individual whose character had been aspersed, but as the representative of one of the committees of the American Medical Association—as a representative of the Association itself. He did not consider the attack a personal, but a national offence, and he did not wish it viewed in any other light. He was charged in that Journal (here he read the article), with having misrepresented the state of the science on which he was called upon to report. He had not done so knowingly or willingly—he had not done so at all. In compiling his report he had examined all the records, had access to the medical journals, had written many letters, had made many inquiries, and he believed that the report was fully up to the state of obstetrical medicine in America. He hoped gentlemen would understand him. He spoke not as Dr. Storer but as chairman of the committee, and he branded the charges made in Dr. Nelson's Journal by some anonymous, but he believed not unknown writer, as unjust, ungentlemanly, ungenerous, and untrue. He begged the pardon of the Association for having consumed so much of its time, and resumed his seat amid great applause.

Dr. Hays, of Philadelphia, arose and said that he too had been mentioned in scurrilous terms in the article just read by his distinguished friend from Boston. He confessed to feeling complimented that his name had been found in such good company—as much complimented by the mention made of so humble an individual as himself as he supposed the writer would be at having been mentioned in the American Medical Association.

The report of the Committee on the Constitution was called for, and Dr. Hays, the chairman, proceeded to read it. Dr. Yardley, a member of the committee made a counter report.

Dr. Atlee, of Lancaster, Pa., spoke in favor of Dr. Hays' report. Had great confidence in the judgment of the distinguished gentleman from Philadelphia—knew no man in the Association better qualified to suggest changes in the constitution than Dr. H.

Moved by Dr. Lopez, of Mobile, that both reports be received, which was carried, but not until after a good deal of discussion between Drs. Atlee, Wood, Cox, Marsh, Stewart, Lopez and Knight.

Dr. Thomas, of Md., moved that the reports be referred to a committee, with instructions to report on the changes proposed.

Dr. Hays objected. He did not believe that Dr. Thomas' plan would answer—thought it better that every one who had a plan should present it—let the whole matter come up at once and then discuss the proposed alterations *seriatim.* For his own part he did not expect to do all in a day—it would require years to get a perfect constitution, perhaps we never would,—he was satisfied that we should never be able to frame a constitution which suited everybody. All that we could hope for was a constitution which worked well in its main features—the details would gradually correct themselves.

Dr. Stewart, of New York, offered an amendment to Dr. Thomas' resolution, to the effect that the committee be instructed to report tomorrow on the reports of the Committee on the Constitution, which was carried.

Dr. Watson, of New York, offered a resolution referring the report of the nominating committee back to said committee, with instructions to report complete on the standing committees and other committees, as may be requisite for providing business for the Association at its next annual meeting.

An invitation from the New York delegation to the Association to hold its next annual meeting in that city, signed by a large number of persons, was read, and Dr. Watson offered a resolution that it be accepted.

Dr. Pope, of St. Louis, objected to this—hoped the Association would stand by the recommendation of the committee.

Dr. Horace Green, of New York, thought the honor due New York —had never held a meeting there—both physicians and people were desirous that the Association should convene there.

Dr. Hooker, of New Haven, said he would be very glad to accept the hand tendered us from St. Louis, but thought it impracticable; he believed it would be strictly a western meeting; and then the Association had recently met in the West.

Dr. Pope replied that, out of the seven meetings held by the Association, six had been on tide water. He was aware that old gentlemen from the East were afraid to travel in the West, because of the danger of steamboat explosions. Western men, however, considered this a puerile objection and altogether futile!

Dr. Drake here followed in a very amusing speech in favor of St. Louis. The question was called, and carried in favor of New York by a majority of eleven votes—the vote standing ninety-one for New York, eighty against it.

Dr. Haxall, of Richmond, offered the following resolution which was adopted:

*Resolved,* That after to-day the Association will hold a morning session from 9 o clock, A. M., to 3½ P. M., and nc afternoon session.

Dr. Drake offered a resolution to be referred to the select committee on the constitution, that students be required to stay to the end of their first course of lectures.

Dr. Hayward, of Boston, read a letter from Dr. Horatio Adams, of Waltham, Mass., regretting that he had been compelled to forego the pleasure of attending the Association, owing to a serious accident, and presenting the report of the committee on the Action of Water on Lead pipes, and the diseases resulting from it—asking the reference of the report to the committee on Publication. The report was received and referred.

A letter was read from the New York Academy of Medicine, protesting against the college *cliniques* as conducted in that city. The Academy alleges that the *material* of the cliniques is frequently drawn from among the patients of the young physicians who are thereby deprived of interesting cases and important surgical operations, and also not unfrequently of fees. Referred to the Committee on Publication.

Dr Corbin, of Va., read the following resolution which he desired to lie on the table for the present:

*Resolved,* That one member from each state represented in this Association, be appointed a delegate to represent it in the Medical Association of Europe, and this committee be requested to visit the hospitals, and report to the next meeting the various improvements in the several branches of science connected with Medical Education, and in the treatment of diseases in foreign countries.

The Association then took a recess till 4½ P. M.

SECOND *DAY*, *Wednesday, May* 5th, 1852.

*Afternoon Session.*—The President, Dr. Wellford, in the chair.

Dr. Drake submitted the following:

*Resolved,* That all papers and reports on scientific subjects shall be read to the Association before the question of their publication shall be decided.

Dr. Wood, of Philadelphia, opposed the resolution. The Association had appointed a committee on publication, and he thought the publication of all communications should be left to them.

Dr. Phelps offered an amendment, which, with the resolution, was laid on the table.

Dr. Condie, of Philadelphia, presented a paper on Chemistry, from a gentleman, not a member of the Association; and Dr. Drake presented a paper by Dr. Wright, of Ohio, on "the influence of the occupation of Daguerreotypists upon their health." Dr. Condie moved that both papers be referred to a select committee. The President appointed Drs. Rogers, Merritt and Dunbar, with instructions to report to morrow.

Dr. Eve, from the committee on nominations, recommended the following officers for the ensuing year:

*For Secretaries:*—Drs. P. C. Gooch, of Va., Edward L. Beadle, of New York.

*For Committee on Publication:*—Drs. Isaac Hays, of Philadelphia, P. C. Gooch, of Va., E. L. Beadle, of New York, Isaac Parrish, of Pa., E. Emmerson, of Pa., D. F. Condie, of Pa., G. W. Norris, of Pa.

*Committee of Arrangements:*—Drs. Campbell Stewart, of N. York, John Watson of New York, Wm. Rockwell, of New York, James R. Wood, of New York, Robert Watts, jr., of New York, Alfred C. Post, of New York, Jno. G. Adams, of New York, H. D. Buckley, of New York.

The report was received and the gentlemen named were unanimously elected officers of the Association for the ensuing year.

Dr. Dickson, of S. C., not being able to attend the meeting of the Association, entrusted the report of the committee on the "Blending and conversion of the types of Fever," to Dr. A. B. Williams, who proceeded to read it. The report was ordered to be printed and referred to the committee on publication.

Dr. Hayward, of Boston, presented and read the report of the committee on the "Permanent and radical cure of Reducible Hernia," which was ordered to be printed and referred to the committee on publication.

I neglected to report that Tuesday afternoon and Wednesday morning, the reports of the committees were called for.

Dr. Lopez, chairman of the committee on "the Epidemic prevalence of Tetanus," stated that he regretted extremely that he was without a report. He had been unsuccessful in every attempt to gather materials for a report. He had addressed one hundred and fifty circulars to the profession in Alabama, and had received only four replies, and none of them contained anything which he could use. He thought, however, that if granted another year, he would be able to make a suitable report concerning this very interesting subject, and he was accordingly continued as chairman of the committee.

Dr. Wood, of Philadelphia, answered the call for the committee on "Diseases of a Parasitic origin," by saying that he had expected the Association to be so crowded with reports that he had neglected to prepare one, but if it was the wish of the Association to continue him on the committee, he would set to work and furnish one for the next meeting. Continued.

The committees on Epidemics were called for. Dr. Hooker, of New Haven, presented his. Dr. Rayburn, of St. Louis, had addressed a letter to the Association asking for further time, which was granted.

The committees for Virginia and North Carolina had distributed four hundred circulars and had received but two answers. Desiring to be continued, leave was granted.

The committees for Kentucky and Tennessee presented their report.

On Tuesday Dr. Drake introduced a resolution to the effect, that the reports of the standing committees should be submitted to other committees to be appointed by the President, and that they should report as to whether the reports of the standing committees should be published. He followed in a long speech in which he exhibited no little feeling, and in turn, was followed by Drs. Hayward, Hooker, and Lopez, who attacked the resolution with warmth and spirit, the latter gentleman with some bitterness. The resolution was lost by an overwhelming majority.

The debates on Thursday I did not attend. Friday morning the Association made an excursion on the Danville railroad, at the polite invitation of the President of the company. Friday afternoon they held a short session and adjourned to meet at a supper given by the Medical Society of Virginia, at the Exchange Hotel.

